# Wood‐Derived, Vertically Aligned, and Densely Interconnected 3D SiC Frameworks for Anisotropically Highly Thermoconductive Polymer Composites

**DOI:** 10.1002/advs.202103592

**Published:** 2022-01-13

**Authors:** Xiaonan Zhou, Songsong Xu, Zhongyu Wang, Liucheng Hao, Zhongqi Shi, Junping Zhao, Qiaogen Zhang, Kozo Ishizaki, Bo Wang, Jianfeng Yang

**Affiliations:** ^1^ State Key Laboratory for Mechanical Behavior of Materials Xi'an Jiaotong University Xi'an 710049 China; ^2^ High Voltage Switchgear Insulation Materials Laboratory of State Grid Pinggao Group Co., Ltd Pingdingshan 467001 China; ^3^ State Key Laboratory of Electrical Insulation and Power Equipment Xi'an Jiaotong University Xi'an 710049 China; ^4^ Department of Mechanical Engineering Nagaoka University of Technology Nagaoka 940−2188 Japan

**Keywords:** anisotropy, epoxy composites, SiC, thermal conductivities, thermal expansions

## Abstract

Construction of a vertically aligned and densely interconnected ordered 3D filler framework in a polymer matrix is a challenge to attain significant thermal conductivity (TC) enhancement efficiency. Fortunately, many biomaterials with unique microstructures can be found in nature. With inspiration from wood, artificial composites can be rationally designed to achieve desired properties. Herein, the authors report a facile and effective approach to fabricate anisotropic polymer composites by biotemplate ceramization technology and subsequent vacuum impregnation of epoxy resin. The hierarchical microstructure of wood is perfectly replicated in the cellular biomass derived SiC (bioSiC) framework by carbothermal reduction. Owing to the anisotropic architecture of bioSiC, the epoxy composite with vertically aligned dense SiC microchannels shows interesting properties, including a high TC (10.27 W m^−1^K^−1^), a significant enhancement efficiency (259 per 1 vol% loading), an outstanding anisotropic TC ratio (5.77), an extremely low coefficient of linear thermal expansion (12.23 ppm K^−1^), a high flexural strength (222 MPa), and an excellent flame resistance. These results demonstrate that this approach is expected to open a new avenue for design and preparation of high performance thermal management materials to address the heat dissipation of modern electronics.

## Graphical abstract

The anisotropic wood microstructure is faultlessly duplicated into biomass derived SiC/epoxy composite by biotemplate ceramization technology and subsequent impregnation of epoxy resin. The composite exhibits a high thermal conductivity of 10.27 W m^−1^ K^−1^ corresponding to a significant enhancement efficiency, and a low coefficient of linear thermal expansion of 12.23 ppm K−1.

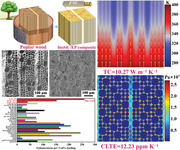

## 1 Introduction

With the rapid development of electronic technology and industry, efficient thermal dissipation has become a stringent necessity in modern electronic devices for stronger performance, higher reliability, and longer lifetime.^[^ ^1^, ^2^, ^3^ ^]^ Thermal management materials has been proven to be the best choice to help address the overheating issue,^[^ ^4^, ^5^, ^6^, ^7^ ^]^ which generally are composed of polymer matrix and inorganic fillers with high thermal conductivity (TC).^[^ ^8^ ^]^ The conventional blending methods have been commonly used to prepare the thermal management materials by randomly dispersing fillers in the polymer matrix.^[^ ^9^ ^]^ However, a large amount of fillers (above 50 vol%) is always needed to attain a continuous and efficient heat conduction path and achieve the target TC values due to the inevitable agglomeration of fillers.^[^ ^10^, ^11^ ^]^ Unfortunately, such a high loading usually leads to high cost, deteriorated mechanical properties and increased processing difficulty.^[^ ^12^ ^]^ Therefore, minimizing the content of fillers while achieving simultaneous high TC to obtain a significant enhancement efficiency remains a great challenge.

As is known, the formation of continuous pathways of fillers is the key to achieve high TC in polymer composites. In recent years, introducing a prebuilt 3D filler network in a polymer matrix has been intensively studied with the purpose of significantly improving the TC enhancement efficiency.^[^ ^13^, ^14^, ^15^, ^16^ ^]^ This thought could ensure that most of the energy is transferred through the 3D filler skeleton, thereby avoiding unnecessary polymer/filler interfacial thermal resistance. In other words, the 3D filler framework act as a macroscopic “motorway” for the rapid transport of phonons throughout the composite, which means that a high TC can be achieved in reduced loading concentration of fillers.^[^ ^17^, ^18^, ^19^, ^20^ ^]^ Hao et al. reported the fabrication of a 3D alumina ceramic skeleton by compression molding and high temperature sintering method, followed by the impregnation of epoxy resin (EP). A remarkable TC of 4.356 W m^−1^K^−1^ in the porous alumina/EP composite was achieved at a filler loading of 43 vol%.^[^ ^21^ ^]^ Tian et al. developed a direct foaming method to prepare a 3D boron nitride porous architecture and then impregnated them with EP. The obtained epoxy composite possesses a high TC of 3.48 W m^−1^K^−1^ at a relatively low filler loading of 24.4 wt%.^[^ ^22^ ^]^ Although the TC of the above composites has been greatly improved compared to those prepared by the conventional methods, the TC enhancement efficiency is still not desirable enough. This is because the isotropic characteristics of these randomly interconnected and homogeneous 3D filler networks mean a zigzag heat conduction path, which was unable to maximize the TC enhancement efficiency. However, if given the anisotropic feature of the 3D filler network, construction of a vertically aligned and densely interconnected 3D filler framework in a polymer matrix is expected to make the heat transfer path straighter, and then further promote the heat conduction throughout the composite.^[^ ^23^ ^]^ In addition, modern electronic substrates also require the materials with low coefficient of linear thermal expansion (CLTE).^[^ ^24^ ^]^ Because the low CLTE substrate materials can reduce thermal evoked stresses and expansion mismatches between substrates and electronic components.^[^ ^25^, ^26^ ^]^ The dense 3D filler skeleton can effectively restrict the thermal expansion of polymer matrix. As an example,^[^ ^27^ ^]^ the CLTE was remarkably decreased by ≈78% for BN/PMMA composite under filling with ≈56.1 wt% BN with respect to pure PMMA, which is ascribed to the embedment of ultralow expansion and tightly interconnected 3D BN skeleton. Meanwhile, the densely interconnected 3D filler framework can carry the load from the polymer matrix, thereby enhancing the mechanical properties of composites.^[^ ^28^, ^29^ ^]^ Zhao et al. used alumina and cyanate ester to creatively synthesize ceramic/polymer composites with 3D interlocking skeleton structure. Such composites exhibit outstanding flexural strength (≈300 MPa), toughness (failure strain ≈5%), and superior specific strength (≈162 MPa (g cm^−3^)^−1^) simultaneously.^[^ ^30^ ^]^ What is more, the anisotropic functionality realized by the anisotropic structure can also expand the range of product applications, such as thermal insulation, thermal dissipation, and directional electrical conduction.

To date, a lot of efforts have been devoted to obtaining an anisotropic 3D filler framework. Among them, the ice‐templated assembly or so called unidirectional freeze‐drying is the most frequent approach owing to its stronger applicability to various materials.^[^ ^31^, ^32^ ^]^ By placing uniform aqueous slurry on the top of a copper pillar which was precooled in liquid nitrogen for unidirectional freezing, the ice crystals will first nucleate at the bottom of the slurry, and then grow along the temperature gradient to form the array of vertically aligned ice columns. At the same time, the fillers are expelled into the gap of adjacent ice columns and gradually templated. A vertically aligned 3D filler network with ordered long range continuous channels is obtained after removing the ice crystals by freeze‐drying.^[^ ^33^, ^34^, ^35^ ^]^ However, the anisotropic factors of 3D filler networks made from bottom–up methods are usually limited, since the pore channels show a poor long range continuity and a large quantity of horizontal branches bridged in the vertical direction.^[^ ^36^ ^]^ In addition, it is difficult for the fillers along the alignment direction to form a dense channel wall by physical extrusion. The existence of these structural defects will inevitably weaken the heat conduction. Moreover, complicated fabrication processes and expensive nano raw materials (such as nanowires, nanosheets, and nanorods) also limit the scaling up of these materials. More rational process design and better selection of materials are required to optimize the anisotropic structure of 3D filler framework and the performance of its composites. Fortunately, many biomaterials with unique microstructures and anisotropy can be found in nature besides this method mentioned above. Millions of years, natural trees have used the vertically aligned cell channels for directional transportation of water and nutrients, and the unique architecture gives rise to anisotropic mechanical and thermal properties in wood.^[^ ^37^, ^38^, ^39^ ^]^ With inspiration from trees, artificial composite materials can be rationally designed to achieve desired properties. Wan et al. prepared a metallic wood with excellent anisotropic electrical, thermal, and mechanical properties by metal continuously filling the wood vessels. The composites boast a high TC of 5.23 W m^−1^K^−1^ at a Sn‐Bi metal filler loading of 31 vol%, and an extraordinary anisotropic TC ratio of 18.^[^ ^40^ ^]^ So far, the exploration of composite materials prepared by natural wood that utilize this anisotropic structure is still limited. Therefore, the development and utilization of structural and functional composites based on inexpensive and environmentally friendly natural wood exhibits strong potential and great significance.

SiC is a promising ceramic filler for polymer composites, which possesses high TC, low CLTE, excellent thermal stability, and remarkable chemical stability.^[^ ^41^, ^42^, ^43^, ^44^, ^45^ ^]^ Generally, the biomass derived carbide ceramics (such as SiC and B_4_C) are fabricated through the following steps: 1) Preparation of carbon templates by pyrolyzing natural materials at high temperature. 2) Infiltration of the carbon template with ceramic precursors (such as liquid silicon, silicon vapor, boron‐containing solution, and silicon‐containing sol). 3) High temperature sintering to form ceramics.^[^ ^46^, ^47^ ^]^ Despite the impressive results reported for biomass derived carbide ceramics synthesized by the existing routes, a few drawbacks still remain unaddressed. The mechanical properties are considerably deteriorated because of the degraded architecture compared with the original natural materials tissue microstructure. Moreover, silicon and boron impurities or residual carbon are always present in the products due to the uncontrollable degree of reaction. Besides it, complicate fabrication process is required for repetitious ceramic precursors infiltration into carbon template.^[^ ^48^, ^49^, ^50^, ^51^ ^]^ In this work, using natural poplar wood as the template, we demonstrate a simple, rapid, and effective approach to fabricate a vertically aligned and densely interconnected 3D biomass derived SiC (bioSiC) skeleton via the carbothermal reduction of SiO vapor with a pyrolytic carbon template and then the impregnation of EP into this framework to obtain the final epoxy composite (bioSiC/EP). The wood tissue anisotropic microstructure is completely replicated in the bioSiC ceramic by employing this wood‐derived technique. As a benefit from this unique architecture, the prepared epoxy composite exhibits a dramatically improved TC (10.27 W m^−1^K^−1^) compared to that of pure EP (0.186 W m^−1^K^−1^) at a relatively low bioSiC loading of 21 vol%, corresponding to a significant enhancement efficiency of 259 per 1 vol% loading. Meanwhile, a high anisotropic TC ratio of 5.77 and an extremely low CLTE of 12.23 ppm K^−1^ are also achieved. These results demonstrate that this method exhibits strong potential for design and preparation of thermal management materials with high heat conduction efficiency. Moreover, we hope this work could provide a new avenue for boosting the development of high performance functional/structural ceramic polymer composites.

## 2 Results and Discussion

**Figure 1:**
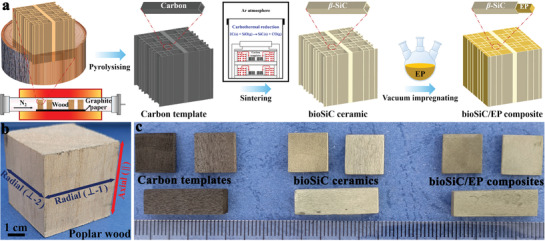
Fabrication and optical images of bioSiC/EP composites. a) Schematic illustration of the preparation process of bioSiC/EP composites. b) Optical image of natural poplar wood as raw materials. c) Optical images of carbon template, bioSiC ceramics, and bioSiC/EP composites.

As schematically illustrated in **Figure** **1**a, the entire procedure of obtaining bioSiC/EP composites consists of three steps: 1) The cut poplar wood was placed in a horizontal tube furnace, which can be converted to the carbon template by pyrolyzing at high temperature under nitrogen shielding. 2) The porous bioSiC ceramics were synthesized by the carbothermal reduction (2C(s) + SiO(g) → SiC(s) + CO(g)) between the carbon templates and silicon monoxide vapor generated from the SiO powders located at the bottom of graphite crucible coated with BN at high temperature under argon atmosphere in a sintering furnace. 3) After surface modification, the functionalized bioSiC ceramics were impregnated with the liquid EP in a vacuum three necked flask followed by thermal curing, forming the final bioSiC/EP composites. Figure 1b shows the digital image of natural poplar wood. As shown in it, the parallel and perpendicular to the tree growth direction was defined as the axial (||) and radial (⊥‐1, ⊥‐2) orientations, respectively. The density of poplar wood was 0.45−0.50 g cm^−3^, a series of volume fractions of the bioSiC ceramic in the bioSiC/EP composite could be obtained based on different density values.

The optical images of carbon template, bioSiC ceramics, and bioSiC/EP composites derived from poplar wood are shown in Figure 1c, which correspond to the schematic diagrams of Figure 1a. A noticeable shape shrinkage was found from poplar wood to carbon template. But the macroscopic characteristics of poplar wood, such as the annual growth rings, were still clearly visible in the carbon template. In contrast to the carbon template, the bioSiC ceramics were no cracks on samples and their bodies were intact with almost no changes in shape and size. but only the body color transformation from black (natural for carbon) to light yellow (natural for SiC) was seen, indicating that the carbothermal reduction reaction occurred. Such a perfect porous bioSiC ceramic template with ordered structure and ultra‐high specific surface area is also suitable for the electrochemical energy storage.^[^ ^52^, ^53^ ^]^ After impregnation with EP, the body color of the bioSiC/EP composites became slightly darker, and its surface looked much smoother and denser. Furthermore, the bioSiC/EP composite could be easily tailored into various shapes and sizes according to the application requirements. The abundant, low cost raw materials and the facile preparation process ensured the scalable fabrication of the bioSiC/EP composite.

**Figure 2:**
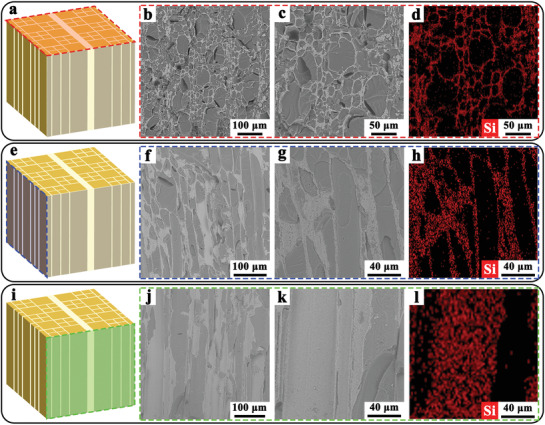
Microstructures of anisotropic bioSiC/EP composite. a) Schematic of bioSiC/EP composite where the red dashed square highlights the cross section perpendicular to the EP alignment direction. b) Corresponding top‐view SEM image of bioSiC/EP composite. c) Enlarged SEM image in (b). d) EDS mapping image of (c). e) Schematic of bioSiC/EP composite where the blue dashed square highlights the cross section parallel to the EP alignment direction (parallel to the cell plate). f) Corresponding left‐view SEM image of bioSiC/EP composite. g) Enlarged SEM image in (f). h) EDS mapping image of (g). i) Schematic of bioSiC/EP composite where the green dashed square highlights the cross section parallel to the EP alignment direction (perpendicular to the cell plate). j) Corresponding main‐view SEM image of bioSiC/EP composite. k) Enlarged SEM image in (j). l) EDS mapping image of (k).

**Figure** **2** presents the microstructures of bioSiC/EP composite. In the schematic shown in Figure 2a, the red dashed square outlines the cross section perpendicular to the EP alignment direction of the bioSiC/EP composite. After high temperature pyrolysis of natural wood, the top‐view SEM image of carbon template shows two kinds of cells. The micropore size of the large ones is in the range of 40−60 µm and that of the small ones is in the range of 10−30 µm with a wall thickness of around 700 nm. In addition, there are some cell plates with a thickness of ≈20–40 µm, which derived from the cell structure of natural wood (Figure S1a, Supporting Information). After carbothermal reduction, the obtained bioSiC ceramic almost completely maintains the microstructure of carbon template (Figure S1d, Supporting Information). Among them, the micropores of carbon template play an important role in effective microstructure inheritance during the conversion from C to SiC. Because these micropores guarantee intimate contacts between amorphous carbon locations inside the carbon template bodies and SiO vapor generated from SiO powders, which leads to spatially homogeneous conversion toward SiC nanocrystals. After surface modification and the EP infiltration, the micropores of bioSiC ceramic are completely filled with the EP. As shown in Figure 2b, the top‐view SEM image of bioSiC/EP composite exhibits different light/dark contrast. The lighter area suggests the bioSiC ceramic, and the darker region is the EP. Figure 2c shows a high‐magnified SEM image of bioSiC/EP composite. A good interfacial adhesion is clearly visible between the bioSiC ceramic and EP, this is due to the favorable compatibility between the modified bioSiC ceramic and EP. The energy‐dispersive X‐ray spectroscopy (EDS) mapping image of bioSiC/EP composite also confirms the composition of the cell wall through the uniform distribution of Si on it (Figure 2d).

The blue dashed square in Figure 2e outlines the cross section parallel to the EP alignment direction of the bioSiC/EP composite (parallel to the cell plate). After high temperature pyrolysis of natural wood, the corresponding carbon template exhibits some smooth aligned cell walls (Figure S1b, Supporting Information). When the C is completely converted to SiC, the bioSiC ceramic still maintains the carbon template microstructure (Figure S1e, Supporting Information). Besides, there are a few micropores at the cell wall of bioSiC ceramic, which were caused by the escape of CO gas from the initial carbon template during the carbothermal reduction. After the EP infiltration, the left‐view SEM image of bioSiC/EP composite shows EP rods aligned and continuously filling the cells in the bioSiC ceramic (Figure 2f). Likewise, Figure 2g shows a high‐magnified SEM image of Figure 2f. The light stripes on the dark EP rods can be clearly seen, demonstrating the intimate contact between the infiltrated EP and the 3D bioSiC network. Figure 2h shows the EDS mapping image of Figure 2g, this indicates that the light stripes are the bioSiC ceramic with the uniform distribution of Si.

The green dashed square in Figure 2i outlines the cross section parallel to the EP alignment direction of the bioSiC/EP composite (perpendicular to the cell plate). After high temperature pyrolysis of natural wood, a dense and smooth cell plate with thickness of about 30 µm can be observed on the carbon template (Figure S1c, Supporting Information). After carbothermal reduction, the bioSiC ceramic retains the cell plate of carbon template (Figure S1f, Supporting Information). After the EP infiltration, the cell plate still exists on the bioSiC/EP composite (Figure 2j). Similarly, Figure 2k shows a high‐magnified SEM image of Figure 2j. The uniform distribution and dense SiC nanocrystals on the cell plate are clearly visible, indicating that the bioSiC ceramic were synthesized by the in situ carbothermal reduction. The EDS mapping image of bioSiC/EP composite also confirm that Si is homogeneously dispersed throughout the cell plate (Figure 2l).

**Figure 3:**
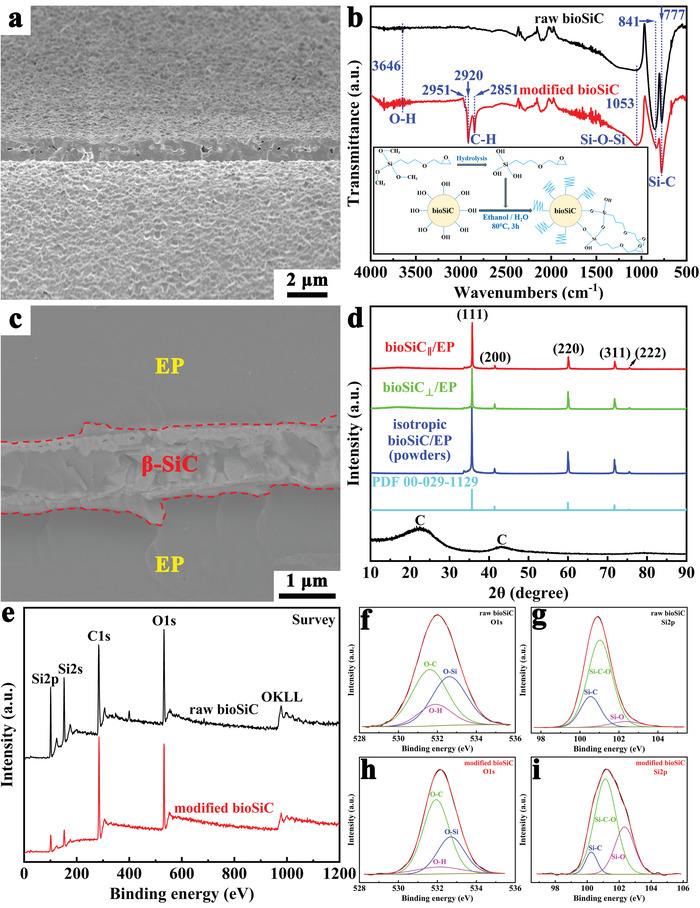
The high magnification SEM images and phase identifications of bioSiC ceramic and bioSiC/EP composite. a) Morphology of the cell wall of bioSiC ceramic. b) FTIR spectra of bioSiC ceramic before and after surface modification. c) Morphology of the cell wall of bioSiC/EP composite. d) XRD patterns of carbon template, *β*‐SiC card, and bioSiC/EP composites. e) XPS survey spectrum of bioSiC ceramic before and after surface modification. XPS high‐resolution XPS spectra of f) O 1s and g) Si 2p peak of raw bioSiC, h) O 1s and i) Si 2p peak of modified bioSiC. The XPS data was pre‐processed by Avantage 5.52, including charge correction and peak fitting.

A high magnification SEM morphology of the cell wall of bioSiC ceramic is shown in **Figure** **3**a. The uniform distribution and fully dense directional columnar SiC nanocrystals on the cell wall are clearly visible. According to our previous research,^[^ ^54^ ^]^ the width and length of SiC nanocrystals are ≈200 and ≈700 nm, respectively. The microstructure inherits the original structural directionality of the cellulose nanofibers of natural wood,^[^ ^55^ ^]^ indicating that the bioSiC ceramics were synthesized by the in situ carbothermal reduction. Moreover, there are a few micropores between the two cell walls of bioSiC ceramic, which were caused by the escape of CO gas from the initial carbon template during the carbothermal reduction. The thickness of the cell wall can be estimated to be about 700 nm, and unreacted carbon is not observed on it, indicating that a complete conversion from C to SiC occurs.

In order to enhance the interfacial adhesion between the EP and 3D bioSiC skeleton, the bioSiC ceramic was modified by silane coupling agent. To demonstrate the effect of modifiers, Fourier transform infrared spectroscopy (FTIR) spectra of the functional groups on the surface of bioSiC ceramic before and after surface modification are characterized in Figure 3b. As shown in it, both the raw bioSiC and modified bioSiC exhibit the peaks at 777 and 841 cm^−1^ for Si–C stretching, and the peak at 3646 cm^−1^ for O–H stretching is one of the features of raw bioSiC surface. After surface modification, the hydroxide group interacted with the hydrolyzed silane coupling agent in ethanol solution, and several functional groups appear on the FTIR spectra. Compared with raw bioSiC, three new absorption peaks at 2851, 2920, and 2951 cm^−1^ are assigned to the valence stretching vibration of aliphatic C–H. Meanwhile, the peak at 1053 cm^−1^ for Si–O–Si stretching is significantly increased, which reflects the dehydration condensation between the silane coupling agents. Unfortunately, the characterizations of O–H and Si–O–Si stretching are not accurate by FTIR spectra, therefore, the X‐ray photoelectron spectrum (XPS )was used to further analyze the bioSiC ceramic before and after surface modification (Figure 3e–i). As shown in Figure 3e, the elementary composition of raw bioSiC and modified bioSiC are primarily Si, C, and O. The O 1s spectrum peak is divided into three peaks at 531.8, 532.1, and 532.7 eV, which are corresponding to O–C, O–H, and O–Si bonds, respectively (Figure 3f,h). Among them, the intensity of the O–H bond is greatly reduced after surface modification, which indicates that the O–H groups of raw bioSiC surface are reacted with the hydrolyzed silane coupling agent. Meanwhile, the Si 2p is fitted by three peaks at 100.4, 101.1, and 102.4 eV, according with Si–C, Si–C–O, and Si–O bonds, respectively. Among them, the intensity of the Si–O–Si bond is significantly enhanced after surface modification (Figure 3g,i), which is due to the dehydration condensation between the hydrolyzed silane coupling agent, this is consistent with the FTIR result. The above results all prove that the EP groups had been successfully coated on the surface of 3D bioSiC network by chemical grafting. To further confirm the effect of surface modification, the high magnification SEM image of the cell wall of bioSiC/EP composite is shown in Figure 3c. The region selected by red dotted line is 3D bioSiC skeleton, and the remaining areas are EP. As shown in it, one can see a good interfacial adhesion between the EP and 3D bioSiC skeleton. This is because the EP groups on the surface of the modified bioSiC can improve the compatibility of bioSiC and EP by forming stronger covalent bonds between the two phases, thereby reducing the interfacial thermal resistance.

X‐ray diffraction (XRD) patterns are used to further characterize the bioSiC/EP composite properties. The two broad and large diffraction bands at approximately 2*θ* = 22.49° and 43.13° are visible in Figure 3d, indicating that the carbon template is composed of amorphous carbon. After carbothermal reduction and vacuum impregnation, the XRD profiles of bioSiC/EP composites all exhibit five characteristic diffraction peaks at approximately 2*θ* = 35.74°, 41.50°, 60.09°, 71.83°, and 75.56°, which is consistent with the (111), (200), (220), (311), and (222) crystal planes of the *β*‐SiC phase card (PDF 00−029−1129), respectively, which confirm the high purity and high crystallinity features of bioSiC ceramic. The analysis of the SEM images from Figure 2 indicates that the bioSiC/EP composite has anisotropy on the microstructure. To further explore its anisotropy on the nanocrystals, XRD characterizations of bioSiC_||_/EP, bioSiC_⊥_/EP, and isotropic bioSiC/EP powders (crushed bulk composite) are performed as shown in Figure 3d. It can be found that there are some differences between the intensity of diffraction peak, which may suggests that the highly orientation of bioSiC nanocrystals in the bioSiC/EP composite.

**Figure 4:**
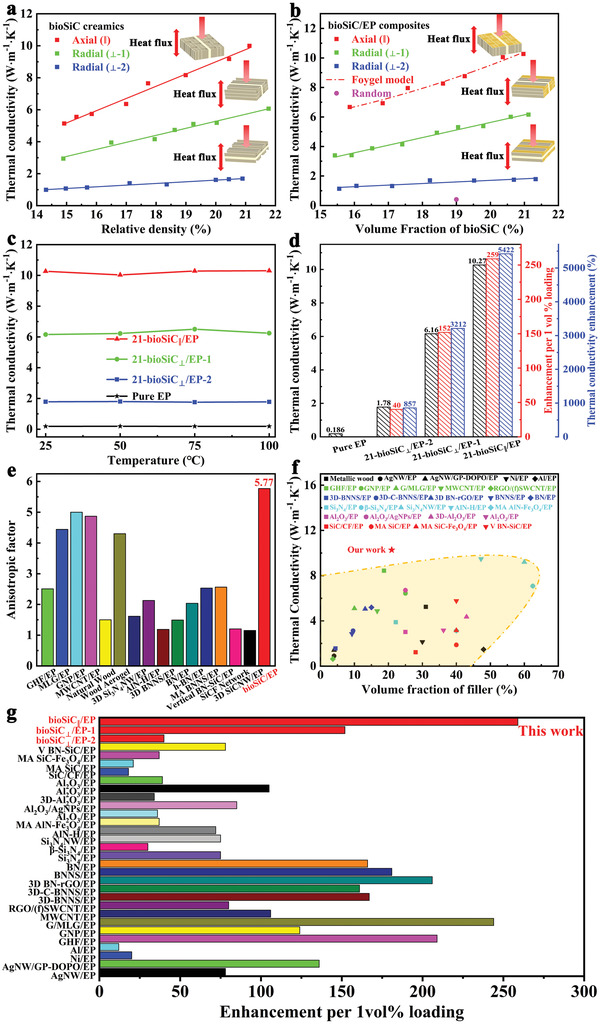
TC of bioSiC ceramics and bioSiC/EP composites. a) Anisotropic TC of bioSiC ceramics as a function of relative density. b) Anisotropic TC of bioSiC/EP composites with different bioSiC loadings. Solid lines in (a,b) were obtained by linear regression of each condition, and dashed line in (b) was obtained by Foygel model of bioSiC_||_/EP composite. According to the fitting, *R* ^2^ values of bioSiC_||_ ceramic, bioSiC_⊥_‐1 ceramic, bioSiC_⊥_‐2 ceramic, bioSiC_||_/EP composite, bioSiC_⊥_/EP‐1 composite, and bioSiC_⊥_/EP‐2 composite are 0.9824, 0.9726, 0.9539, 0.9680, 0.9850, and 0.8293, respectively. c) TC of 21‐bioSiC/EP composites and pure EP as a function of test temperature. d) Anisotropic TC, TC enhancement, and TC enhancement efficiency of 21‐bioSiC/EP composites. Comparison of e) anisotropic TC ratio, f) TC, and g) TC enhancement efficiency of 21‐bioSiC/EP composites and other epoxy composites reported in previous work.

To investigate the correlation between anisotropic microstructure and TC of the bioSiC ceramics and the bioSiC/EP composites, the TC measurements of axial (TC_||_) and radial (TC_⊥_‐1, TC_⊥_‐2) were carried out. **Figure** **4**a shows the variation in TC for the bioSiC ceramics as a function of relative density. With the increasing the relative density of bioSiC ceramics, the TC values of three orientations both show a significant enhancement. When the relative density increases from ≈14% to ≈22%, the TC_||_ of bioSiC ceramics increases from 5.14 to 9.99 W m^−1^K^−1^, the TC_⊥_‐1 increases from 2.94 to 6.06 W m^−1^K^−1^, and the TC_⊥_‐2 increases from 0.99 to 1.69 W m^−1^K^−1^, respectively, which mainly benefited from the increasing number of heat transfer pathways. Moreover, low porosity results in the reduction of phonon scattering, which will be beneficial to phonon transport, and vice versa. On the other hand, the unique anisotropic microstructure of bioSiC ceramics results in an anisotropic TC, the TC_||_ is superior to that of the TC_⊥_, and the TC_⊥_‐1 is higher than the TC_⊥_‐2.

**Figure 5:**
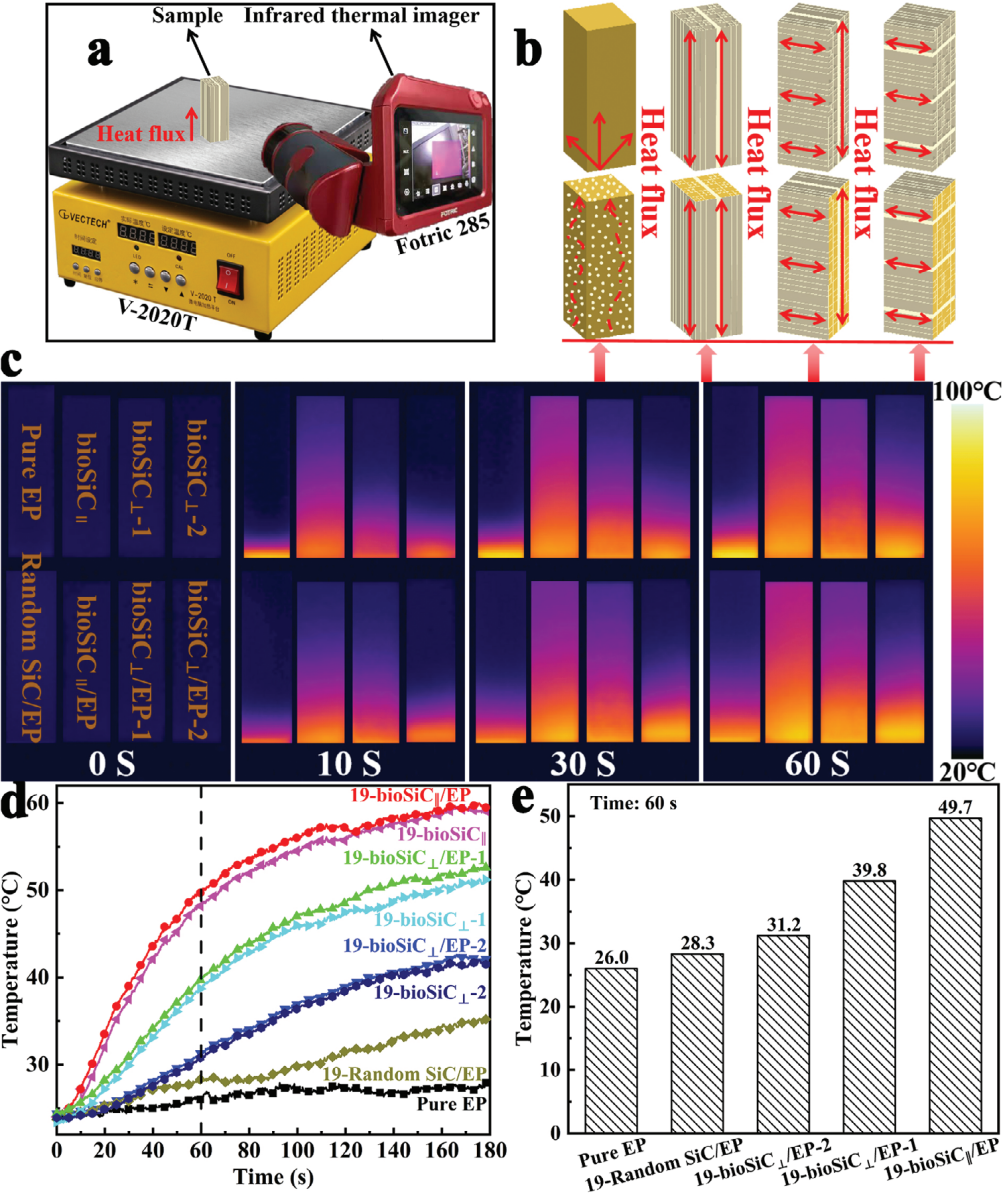
Thermal management applications of pure EP, bioSiC ceramics with 81% porosity, random SiC/EP, and bioSiC/EP composites with 19 vol% bioSiC. a) Experimental device for infrared thermal imaging. b) Schematic illustration of the heat conduction mechanism in pure EP, bioSiC ceramics, random SiC/EP, and bioSiC/EP composites. c) Corresponding infrared thermal images of pure EP, bioSiC ceramics, random SiC/EP, and bioSiC/EP composites at different heating times. d) Top surface temperature variations with heating time of pure EP, bioSiC ceramics, random SiC/EP, and bioSiC/EP composites. e) Top surface temperatures at 60 s of pure EP, random SiC/EP, and bioSiC/EP composites.

Comparing with the bioSiC ceramics, after the EP infiltration, the TC of bioSiC/EP composites exhibits a similar trend (Figure 4b). The TC of bioSiC/EP composites increases linearly with the filler loading, indicating that most of the introduced 3D bioSiC network contribute to the formation of continuous thermally conductive pathways in the composites. As shown in **Figure** **5**b, the TC_||_ of 19‐bioSiC/EP composite is about 20 times greater than that of random SiC/EP composite (0.41 W m^−1^K^−1^) with same filler loading, which indicated that the vertically aligned bioSiC microchannels in EP matrix serve as a phonon “expressway” to facilitate thermal conductive in composites. At the same filler loading (≈21 vol%), the TC_||_ of bioSiC/EP composites slightly increases from 9.99 to 10.27 W m^−1^K^−1^, the TC_⊥_‐1 increases from 6.06 to 6.16 W m^−1^K^−1^, and the TC_⊥_‐2 increases from 1.69 to 1.78 W m^−1^K^−1^, respectively. This is because the bioSiC ceramics are equivalent to bioSiC/air composites, the TC of pure EP (0.186 W m^−1^K^−1^) is slightly higher than that of air (0.026 W m^−1^K^−1^). Therefore, the values of bioSiC/EP composites exhibit slight increase comparing with the TC of the bioSiC ceramics. These results indicate that the TC of bioSiC/EP composites is mainly provided by the 3D bioSiC framework, and most heat is quickly transferred through bioSiC. Therefore in our composites, the interfacial thermal resistance is mainly originated from bioSiC‐bioSiC interface not bioSiC–EP interface. To calculate the interface thermal resistance, as exhibited in Figure 4b, a nonlinear model proposed by Foygel et al. is applied to the bioSiC_||_/EP composite (Equation S1, Supporting Information).^[^ ^56^ ^]^ Some parameter values obtained by perfectly fitting the experimental data can be used to calculate the contact resistance (*R*) between adjacent bioSiC (Equation S2, Supporting Information). The *R* of bioSiC_||_/EP composite can be estimated as 3.49 × 10^5^ K W^−1^, which is 1–3 orders of magnitude smaller than the BNNS/EP composite prepared by freeze‐drying (Table S1, Supporting Information).^[^ ^31^, ^57^ ^]^ This qualitatively explains why the bioSiC/EP composites have higher TC compared with other epoxy composites prepared by freeze‐drying. Because beside the 3D continuous skeleton structure, the density of framework is another key factor for phonon transport. The 3D bioSiC network prepared by sintering process could bring densely connected bioSiC junctions, which possess much stronger interaction and lower *R* than covalent bonds.^[^ ^41^ ^]^ Figure 4c shows the temperature‐dependent TC of bioSiC/EP composites at a filler loading of 21 vol% and pure EP. The TC exhibits a slight change (±5%) between 25 and 100 ℃, suggesting stable capability of heat conduction in this temperature range. Such a small variation of TC with temperature would be beneficial for long term device operation.

In addition, since the anisotropic microstructure of 3D bioSiC skeleton, the TC of bioSiC/EP composites also shows the anisotropic nature. The anisotropic TC, TC enhancement, and TC enhancement efficiency of bioSiC/EP composites with 21 vol% bioSiC are illustrated in Figure 4d. The pure EP has a low TC of 0.186 W m^−1^K^−1^ at room temperature, which agrees well with previous results.^[^ ^31^, ^32^, ^58^ ^]^ The existence of 3D bioSiC structure results in a dramatic enhancement of TC of the EP. The TC_||_ of bioSiC/EP composites achieves a high value of 10.27 W m^−1^K^−1^ at a low bioSiC content of 21 vol%, corresponding to 5422% TC enhancement compared with pure EP. But the TC_⊥_‐2 can only reach to 1.78 W m^−1^K^−1^ at the same loading fraction, which is equivalent to 857% TC enhancement of pure EP. The large TC difference between these two orientations is mainly attributed to the straighter and wider heat transfer pathway in bioSiC_||_/EP composite compared to the tortuous and complicated heat transfer pathway in bioSiC_⊥_/EP‐2 composite. The above results suggest the important role of constructing a vertically aligned and densely interconnected 3D filler framework on attaining the significant TC enhancement at a lower filler loading for the composite. In order to further exhibit the superiority of the 3D bioSiC skeleton on the TC enhancement of composite, the TC enhancement efficiency (*η*, enhancement per 1 vol% loading) is calculated. Here, *η* is defined as:

(1) $$\eta =\frac{K-K_{m}}{100V_{f}K_{m}}\times 100\%$$

where *K* and *K*_m_ are the TC of the bioSiC/EP composites and pure EP, respectively, and *V*_f_ is the volume fraction of bioSiC in composites. As shown in Figure 4d, the *η* values of three orientations of the bioSiC/EP composites reach 40, 152, and 259 at a filler loading of 21 vol%, respectively.

Many applications can benefit from the excellent anisotropic thermal properties of epoxy composite.^[^ ^59^, ^60^, ^61^ ^]^ As shown in Figure 4e, the anisotropic TC ratio (TC_||_/TC_⊥_‐2) in the bioSiC/EP composites is 5.77, which is much higher than that of natural wood (1.50), even higher than that of wood aerogel (4.30) with layered structure after chemical treatment. Benefiting from this anisotropic architecture, the anisotropic factor of bioSiC/EP composites is over 5 times higher than other SiC/EP composites. In addition, most of the reported epoxy composites fall in the anisotropic TC ratio range of 1.00–3.00, where the graphite nanoplatelet epoxy composite exhibits a relatively better performance with an anisotropic factor of 5.00, which is still less than the bioSiC/EP composite. For the epoxy composites prepared by freeze drying, their anisotropic TC ratios are usually relatively low due to poor long range continuity, such as graphite epoxy composites (2.51–4.44), CNT epoxy composite (4.87), Si_3_N_4_ epoxy composite (1.61), AlN epoxy composite (2.13), BN epoxy composites (1.19–2.56), and SiC epoxy composite (1.15) (Table S2, Supporting Information).^[^ ^17^, ^22^, ^31^, ^41^, ^62^, ^63^, ^64^, ^65^, ^66^, ^67^, ^68^, ^69^, ^70^, ^79^ ^]^ In addition, we also compared the TC of the bioSiC/EP composites with other epoxy composites reported in previous work (Figure 4f), including metallic, graphite, BN, Si_3_N_4_, AlN, Al_2_O_3_, and SiC epoxy composites (Table S3, Supporting Information).^[^ ^21^, ^22^, ^23^, ^31^, ^32^, ^40^, ^58^, ^62^, ^65^, ^67^, ^70^, ^71^, ^72^, ^73^, ^74^, ^75^, ^76^, ^77^, ^78^, ^79^, ^80^, ^81^, ^82^, ^83^, ^84^ ^]^ A high TC_||_ of 10.27 W m^−1^K^−1^ of the bioSiC/EP composites with 21 vol% bioSiC is achieved. The TC_||_ is considerably higher than other epoxy composites at the same filler loading (≈21 vol%), which is even higher than some epoxy composites with high filler loading (>50 vol%). In addition, most of the reported epoxy composites fall in the TC range of 1.00–6.00, where the AlN honeycomb epoxy composite exhibits a relatively better performance with a TC of 9.48 W m^−1^K^−1^ at a filler loading of 47.26 vol%, which is still less than the TC_||_. On the other hand, the TC_||_ is much larger than that of SiC epoxy composites prepared by other methods. The *η* values of some typical thermally conductive epoxy composites are summarized in Figure 4g, and the detailed data are listed in Table S3, Supporting Information.^[^ ^21^, ^22^, ^23^, ^31^, ^32^, ^58^, ^62^, ^65^, ^67^, ^70^, ^71^, ^72^, ^73^, ^74^, ^75^, ^76^, ^77^, ^78^, ^79^, ^80^, ^81^, ^82^, ^83^, ^84^, ^85^ ^]^ Most of the reported epoxy composites fall in the *η* range of 10−200, where the graphene‐multilayer graphene epoxy composite exhibits a relatively better performance with a *η* value of 244. It is worth noting that the vertically aligned and densely interlinked 3D bioSiC framework exhibits the highest *η* value of 259 among the reported fillers. There is no doubt that our bioSiC/EP composites simultaneously exhibit the highest TC and efficiency, indicating that the above structure has the better capability to improve heat transfer performance of epoxy composites.

In order to visually demonstrate the heat conduction performance of the samples, the pure EP, three orientations of bioSiC ceramics with 81% porosity, random SiC/EP and three orientations of bioSiC/EP composites with 19 vol% bioSiC with dimensions of 20 mm × 6 mm × 6 mm were successively placed on the same heating platform at 100 ℃ for heating, during which their surface temperature variations were recorded by an infrared thermal imager, as shown in Figure 5a.

To better understand the heat conduction mechanism of the 3D bioSiC skeleton, combined with the microstructures and TC results of samples, and the thermal transfer models of pure EP, bioSiC ceramics, random SiC/EP, and bioSiC/EP composites are proposed, as illustrated in Figure 5b. The EP is difficult to form effective thermally conductive networks due to the strong phonon scattering of the randomly entangled molecule chains,^[^ ^9^ ^]^ thereby the phonons need considerable time to pass through the EP matrix, resulting in a low TC of 0.186 W m^−1^K^−1^ (Figure 4d). Similarly, the uniformly dispersed SiC particles cannot form continuous heat conduction pathways in the random SiC/EP composite. The thermal transfer among SiC particles is difficult due to large contact thermal resistance between bioSiC–EP interface. For the bioSiC/EP composites, the 3D bioSiC frameworks act as a macroscopic “motorway” for the rapid transport of phonons throughout the composite. Obviously, the best way to cross the EP matrix is to build a straighter and wider heat transfer pathway, as shown in bioSiC_||_/EP composite. The phonons transport unidirectionally along the aligned cell wall and cell plate of bioSiC_||_ ceramic, which have almost no scattering. Therefore, the TC of bioSiC_||_/EP composite is greatly improved compared to pure EP. By contrast, for the bioSiC_⊥_/EP‐1 composite, the heat flows along the cell plate and perpendicular to the cell wall of bioSiC_⊥_‐1 ceramic. As a result, some phonons need to jump from one highway to another to cross the EP matrix, and it will take a relatively long time and produce a relatively large interface thermal resistance. For the bioSiC_⊥_/EP‐2 composite, the heat flows along perpendicular to both the cell plate and cell wall of bioSiC_⊥_‐2 ceramic. There is no straight heat transfer pathway, and the considerable interfacial thermal resistance is generated because of the greater number of gaps, thereby leading inevitably to a low TC.

The corresponding infrared thermal images with time are shown in Figure 5c, for the bioSiC ceramics, it is observed that the surface temperature of bioSiC_||_ ceramic increases with time much faster than other samples, followed by the bioSiC_⊥_‐1 ceramic, whose surface temperature shows a slightly faster increase than that of bioSiC_⊥_‐2 ceramic. In addition, the bioSiC/EP composites display the same trend, but they are much faster than that of the EP, which is consistent with the TC of these samples. It is worth noting that, the surface temperatures of two samples with the same orientation of bioSiC ceramics and bioSiC/EP composites increase at almost the same rate due to their similar TC. Such character indicates again that the heat transfer capacity of bioSiC/EP composites is almost completely provided by the 3D bioSiC networks. Although the surface temperature of random SiC/EP composite increases with time slightly faster than pure EP, which is still much smaller than bioSiC/EP composites. The results are also confirmed by the top surface temperature‐heating time curves that can be observed in Figure 5d. As shown in Figure 5e, after heating for 60 s, the top surface temperature of 19‐bioSiC_||_/EP composite is very close to 50 ℃, but the temperature of other samples is always much lower than 50 ℃. Especially the pure EP and 19‐random SiC/EP composite, the temperatures are only 26 and 28.3 ℃, respectively, which are close to room temperature. The above results show that the bioSiC/EP composites have huge potential for anisotropic thermal management applications.

**Figure 6:**
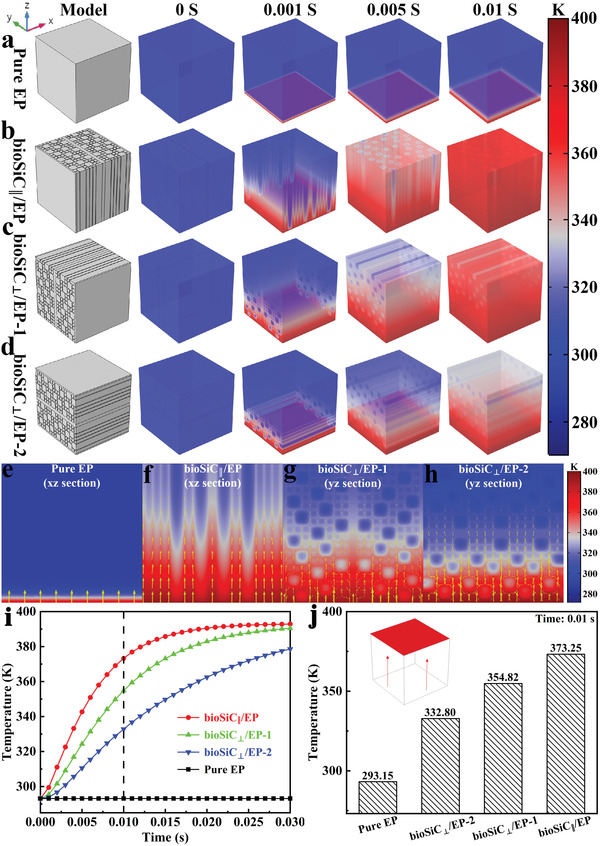
Heat transfer process simulated by finite element simulation. Temperature distribution of a) pure EP, b) bioSiC_||_/EP composite with bioSiC volume ratio of 20%, c) bioSiC_⊥_/EP‐1 composite with bioSiC volume ratio of 20%, and d) bioSiC_⊥_/EP‐2 composite with bioSiC volume ratio of 20%. Temperature distribution and heat flow arrows of e) the section of (a) at 0.003 s, f) the section of (b) at 0.003 s, g) the section of (c) at 0.003 s, and h) the section of (d) at 0.003 s. i) Top surface temperature variations with heating time of pure EP and bioSiC/EP composites. j) Top surface temperatures at 0.01 s of pure EP and bioSiC/EP composites.

In order to further study the thermal conduction behavior of the bioSiC/EP composites, the finite element simulation was carried out using COMSOL Multiphysics 5.4. As shown in **Figure** **6**, heat transfer process of pure EP and three orientations of the bioSiC/EP composites at 20 vol% bioSiC were simulated. First, four kinds of models with the same size of 384 × 384 × 384 µm^3^ were created. Then, physics field and boundary conditions were added. All the boundaries of model except for the bottom have an initial value at room temperature (20 ℃), and the temperature at the bottom is fixed to 120 ℃, while all other outer boundaries are perfectly thermal insulated. Finally, transient finite element analysis methodology was established, the total time length is 0.03 s with a step length of 0.001 s. All the four models were set as the same conditions, when the bottom surface is exposed to a constant temperature heat source (120 ℃), convection is applied to the peripheral surfaces and the thermal conduction performance from the bottom to the top surface can be evaluated.

Figure 6a–d shows the temperature distribution of pure EP and three orientations of the bioSiC/EP composites over time, and the dynamic variations are shown in Figure S2, Supporting Information. The distribution temperature represents the heat transfer speed in the materials. For pure EP, the heat transfers uniformly and slowly from the bottom to the top as a result of its low TC (0.186 W m^−1^K^−1^), while in bioSiC/EP composites, the heat transfers much faster from one side to another. It is worth noting that the bioSiC_||_/EP composite exhibits a faster thermal transfer with time in comparison with the bioSiC_⊥_/EP‐1 composite, but much faster than that of the bioSiC_⊥_/EP‐2 composite. The top surface temperature recorded in Figure 6i can also prove the above results. The top surface temperature of the pure EP remain no change in the whole process, while for bioSiC/EP composites, they get close to the temperature of the bottom quickly. In addition, it can be seen that the top surface temperature of bioSiC_||_/EP composite increases faster compared to bioSiC_⊥_/EP composite from the curves. The top surface temperature values of pure EP and three orientations of the bioSiC/EP composites are 20.00, 59.65, 81.67, and 100.10 ℃ at 0.01 s, respectively (Figure 6j). This is consistent with the test results of TC and infrared thermal imaging.

To further illustrate the heat transfer process, the temperature distribution and heat flow arrows of the sections of four models at 0.003 s are shown in Figure 6e–h. Combined with the heat flow arrows, heat flows equally from each region for pure EP. But for bioSiC/EP composites, the heat flow arrows are mainly concentrated in the bioSiC region, indicating that most heat is quickly transferred through 3D bioSiC network due to its high TC (300 W m^−1^K^−1^). For bioSiC_⊥_/EP‐2 composite as shown in Figure 6h, where the input heat flow is perpendicular to the EP alignment direction and the cell plate. The heat is transferred upward layer by layer along the direction of perpendicular to the cell wall of bioSiC from the bottom, and the pathway is tortuous and complicated. As a result, the majority of input heat is dissipated horizontally before reaching the top surface, resulting in a relatively low temperature on the top surface. And for bioSiC_||_/EP composite as shown in Figure 6f, where the input heat flow is parallel to the EP alignment direction and the cell plate. The heat is directly and quickly transferred upward through the cell wall of bioSiC from the bottom, and the pathway is straighter and wider. While for bioSiC_⊥_/EP‐1 composite as shown in Figure 6g, where the input heat flow is perpendicular to the EP alignment direction and parallel to the cell plate. Therefore, the heat transfer rate is between the bioSiC_⊥_/EP‐2 composite and the bioSiC_||_/EP composite. In summary, the more direct and simple the heat transfer pathway is, the more efficient the pathway is, and the faster the heat transfer will be. Based on the parallel relationship between the microchannel alignment direction (including the cell plate) and the heat flow direction, the thermal interface material with anisotropic heat transfer performance can be easily customized.

**Figure 7:**
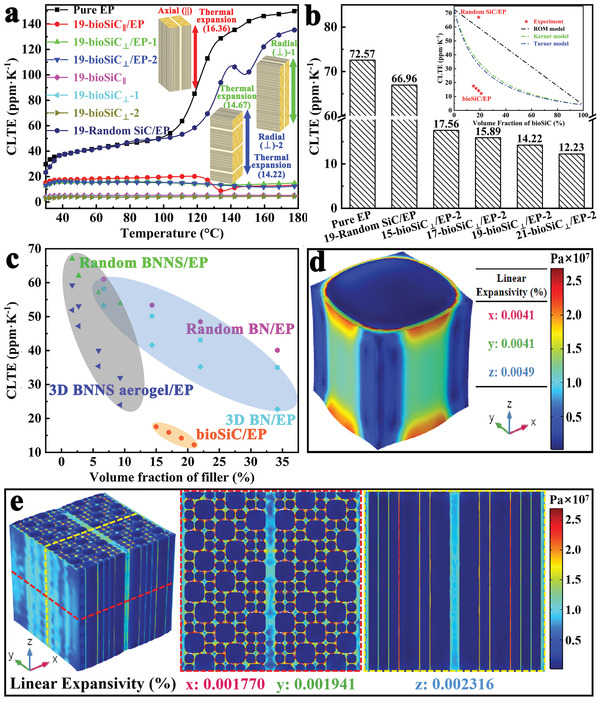
CLTE of pure EP, bioSiC ceramics, random SiC/EP, and bioSiC/EP composites. a) CLTE curves as a function of temperature. b) CLTE values of bioSiC/EP composites with different bioSiC loadings. c) Comparison of CLTE values of bioSiC/EP composites and other epoxy composites reported in previous work. The CLTE values were the average CLTE in the temperature range of 30–180 ℃. Thermal expansion process of bioSiC/EP composites simulated by finite element simulation. Thermal stress distribution and linear expansivity of d) a single unit and e) multiple units with bioSiC volume ratio of 20% at 120 ℃.

The dimensional stability is an essential property for polymer composites, because the poor dimensional stability would bring severe impacts to the reliability of electronic devices.^[^ ^31^, ^86^ ^]^ The CLTE value is indicative of the dimensional stability, and is tested via thermo‐dilatometry measurements within the temperature range of room temperature to 180 ℃. The CLTE curves of pure EP, bioSiC ceramics, random SiC/EP, and bioSiC/EP composites as a function of temperature are shown in **Figure** **7**a. For pure EP, the CLTE monotonically increases with temperature. It is obvious that the value increased the fastest at ≈120 ℃ (near to that of the glass transition temperature), which is due to the polymer chain motion state changes and thus leads to increase in the segmental mobility. The CLTE of random SiC/EP composite with 19 vol% SiC is slightly lower than that of pure EP, and is much lower than that of bioSiC/EP composites with same filler loading, which indicated that the separate distributed SiC particles cannot restrict the segmental mobility of polymer chain. While for bioSiC ceramics with 19% relative density, the CLTE of three orientations both remains almost unchanged in the whole process, and has a very low value of ≈4 ppm K^−1^. For bioSiC/EP composites with 19 vol% bioSiC, there are some slight fluctuations in the CLTE curves of three orientations with the variation of temperature, but the overall change is small. It could be seen that the CLTE of bioSiC/EP composites are much less than the curve of pure EP and close to the bioSiC ceramics. This is attributed to the well aligned 3D bioSiC structure stabilizing the whole framework of the composites. In addition, after further calculation, the CLTE values of three orientations of the bioSiC/EP composites reach 16.36, 14.67, and 14.22 ppm K^−1^ at a filler loading of 19 vol%, respectively. It is worth noting that the bioSiC/EP composites exhibit anisotropic CLTE in the axial and radial, and the CLTE values of bioSiC_⊥_/EP composites are lower than the bioSiC_||_/EP composite.

The effect of bioSiC loading on CLTE values of bioSiC/EP composites is discussed in Figure 7b. As shown in it, the CLTE value of pure EP is as high as 72.57 ppm K^−1^. With the increasing addition of bioSiC, the CLTE values of bioSiC/EP composites show a significant decrease, which mainly because the 3D bioSiC framework exerts a stronger confinement effect on the motion of EP molecular chains. And the higher the volume fraction of bioSiC, the more obvious the inhibition. The minimum CLTE value of 12.23 ppm K^−1^ in bioSiC/EP composites is achieved with 21 vol% bioSiC, which is about 5 times lower than that of the pure EP. To further explore the effect of 3D bioSiC skeleton on CLTE, three classical models including Rule of Mixture (ROM), Turner, and Kerner are applied to estimate the theoretical CLTE values of bioSiC/EP composites (Equations S3–S5, Supporting Information).^[^ ^87^, ^88^ ^]^ As shown in the inset of Figure 7b, the CLTE value of 19‐random SiC/EP composite is 66.96, which is close to ROM model. The experimental values of bioSiC/EP composites are much smaller than the calculated values from the models. This is because these three models are to derive the thermal expansion of a composite with zero internal stress and phase constitutions with similar modulus. And for our composites, the large difference in CLTE value between 3D bioSiC network and EP matrix leads to the existence of internal stress, which contributes to improvement of the polymer system stability. To accurately evaluate the thermal properties of the 3D bioSiC framework reinforced epoxy composites, Figure 7c illustrates the comparison of CLTE values of the bioSiC/EP composites with other BN/EP composites reported in previous work.^[^ ^31^, ^36^ ^]^ Both the 3D and random comparison samples show a tendency to decrease with extended filler loading. 3D filler network/EP composites present a lower CLTE, which attribute this reduction to the suppression of the volume change in the 3D structure between filler and EP in the interstitial space. In addition, the bioSiC/EP composites possess a lowest CLTE value of 12.23 ppm K^−1^, whereas the lowest values of BNNS aerogel/EP composites and oriented BN/EP composites are 24.00 and 22.70 ppm K^−1^, respectively. This consequence indicates that the vertically aligned and densely interlinked 3D bioSiC network with latticed structure exerts a stronger inhibition effect on the thermal expansion of EP.

In order to further explain the anisotropy and internal stress of bioSiC/EP composites during thermal expansion process, the finite element simulation was carried out using COMSOL Multiphysics 5.4. The models of a single unit and multiple units with bioSiC volume ratio of 20% were created, respectively, the temperature of both models was fixed to 120 ℃. Figure 7d shows the thermal stress distribution and linear expansivity of a single unit, it can be seen that the thermal stress is the largest at the interface between 3D bioSiC framework and EP. According to the average displacement of each surface of the model in Table S4, Supporting Information, the linear expansivity in all directions can be calculated. The linear expansivity of the single unit in the *z* direction (0.0049%) is higher than that in the *x* and *y* directions (0.0041%). While for the model of multiple units, the same result can be observed in Figure 7e. Although the linear expansivity of the bioSiC/EP composites in the through‐plane (*z* direction) is as low as 0.002316%, it is still higher than the in‐plane (*x* direction: 0.001770%, *y* direction: 0.001941%). From the section figures, it is obvious that the large thermal stress is still concentrated at the interface. In the in‐plane direction, the thermal expansion of EP is inhibited by the cell wall of bioSiC under normal stress, and the force of each cell wall is superimposed. Thus, the average displacement of the corresponding surface is relatively small. While in the through‐plane direction, the thermal expansion is restrained by the cell wall of bioSiC under shear stress. By contrast, the force of each cell wall is independent, thereby the average displacement of the top and bottom surface is relatively large. This result illustrates again that the bioSiC/EP composite is anisotropic during thermal expansion process, the linear expansivity of bioSiC_||_/EP composite (through‐plane) is higher than bioSiC_⊥_/EP composite (in‐plane).

**Figure 8. a):**
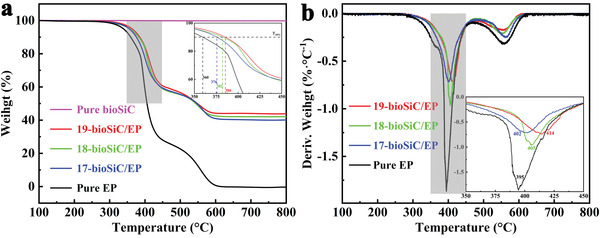
TGA and b) DTG curves of the pure bioSiC and EP and the bioSiC/EP composites with different SiC loading at air atmosphere.

Thermal stability is an important consideration of thermal management materials, the limiting factor in being both production and applications.^[^ ^89^ ^]^ Thermogravimetric tests were carried out to investigate the thermal stability of the bioSiC/EP composites (**Figure** **8**). As shown in Figure 8a, pure bioSiC exhibits no weight loss up to 800 ℃, indicating their remarkable resistance to oxidation, which is in agreement with previous reports.^[^ ^43^ ^]^ However, the thermal gravimetric analyzer (TGA) curves of pure EP and bioSiC/EP composites all have two stage degradation behavior at 270−470 and 470−620 ℃, which correspond to the decomposition of the oxygen‐containing groups on the edge of EP and the pyrolysis of the carbon–carbon skeleton, respectively. Accordingly, the weight of bioSiC/EP composites at 620−800 ℃ can been used to characterize the content of bioSiC. The temperature at 10 wt% loss of the samples (*T*_10%_) provides vital information about thermal stability. As shown in the inset of Figure 8a, the pure EP exhibits a *T*_10%_ of 360 ℃, as the bioSiC loading increases from 17 to 19 vol%, the *T*_10%_ increases from 376 to 386 ℃, which indicates that the addition of bioSiC can retard the EP degradation, and the thermal stability of bioSiC/EP composites increases with increasing the loading fraction of bioSiC. It can be explained from the following three points: 1) The bioSiC exhibits higher thermal stability than EP. 2) The bioSiC possesses higher TC than EP, which makes it easier to absorb heat and results in the degradation of EP at higher temperature. 3) The 3D bioSiC framework possesses a barrier effect, which can inhibit the mobility of EP, delay the escape of volatile degradation products and prevent the transfer of oxygen.

As shown in Figure 8b, the addition of bioSiC results in a shift of the peak of DTG curve to a higher temperature compared to pure EP (395 ℃). Moreover, the temperature at the maximum decomposition rate of bioSiC/EP composites exhibits an increase with increasing the bioSiC loading. The maximum temperature of 414 ℃ in bioSiC/EP composites is achieved with 19 vol% bioSiC, which is nearly 20 ℃ higher than that of the pure EP. The information detail of the thermal stability is shown in Table S5, Supporting Information. This property benefits longer lifetime when the composites are used as thermal interface materials.

**Figure 9:**
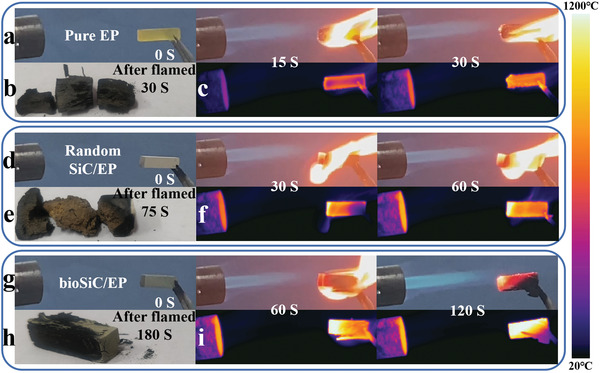
Flame resistance test of pure EP, random SiC/EP, and bioSiC/EP composites. Optical images of a) pure EP, d) random SiC/EP, and g) bioSiC/EP composites at different flaming times. Optical images of b) pure EP, e) random SiC/EP, and h) bioSiC/EP composites after flamed. Infrared thermal images of c) pure EP, f) random SiC/EP, and i) bioSiC/EP composites at different flaming times.

Flame retardancy is an important factor to ensure its fire safety during use for thermal management materials.^[^ ^59^, ^60^, ^90^ ^]^ Therefore, the flame resistance properties of pure EP, random SiC/EP, and bioSiC/EP composites were characterized by a butane spray gun (**Figure** **9**). As shown in Figure 9a, the pure EP was combustible and broke into black blocks at 30 s when it was close to the flame (Figure 9b). For the random SiC/EP composite, the burning time had been extended compared with that of pure EP (Figure 9d), indicating that the existence of SiC particles effectively delayed the diffusion of flame. However, the whole composite eventually broke up at 75 s with the combustion of EP matrix (Figure 9e). Whereas, for the bioSiC/EP composite, as shown in Figure 9g, the flame went out when the EP matrix burnt out. It is worth noting that the bioSiC/EP composite could maintain its initial shape after burning for 180 s (Figure 9h), which is due to the densely interconnected 3D bioSiC structure stabilizing the whole framework of the composite.^[^ ^90^, ^91^, ^92^ ^]^

Combined with infrared thermal images (Figure 9c,f,i), the pure EP showed obvious deformation after burning for 30 s, however, the random SiC/EP and bioSiC/EP composites could maintain its initial shape. In addition, the surface temperature of bioSiC/EP composite is higher than the random SiC/EP composite at the same combustion time (60 s), indicating that the heat transfer is faster in the bioSiC/EP composite. This result is consistent with the infrared imaging result (Figure 5c). Therefore, such an outstanding flame retardant performance enables the bioSiC/EP composite to serve as a promising thermal management material for thermal manipulation at high temperature as well as validating practical work ability in some extreme conditions, such as fire circumstances.^[^ ^91^, ^92^ ^]^

**Figure 10:**
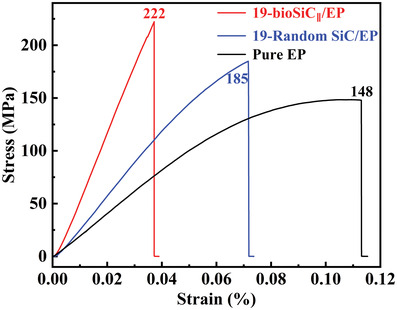
Flexural strengths of pure EP, random SiC/EP, and bioSiC/EP composites. Detailed information is described in the Experimental Section.

**Figure** **10** shows the stress–strain curves of pure EP, random SiC/EP and bioSiC/EP composites. The flexural strengths of pure EP, random SiC/EP, and bioSiC/EP composites are 148, 185, and 222 MPa, respectively. The flexural strength of random SiC/EP composite is slightly higher than the pure EP. While for the 3D‐bioSiC/EP composite, its flexural strength is increased by 50% compared to the pure EP, indicating that the densely interconnected 3D bioSiC framework can carry the load from the EP matrix,^[^ ^28^, ^29^ ^]^ thereby enhancing the mechanical properties of epoxy composites.

## 3 Conclusion

In summary, using natural poplar wood as the template, bioSiC/EP composite with excellent thermal properties was successfully prepared by constructing a vertically aligned and densely interconnected 3D bioSiC skeleton via the carbothermal reduction and subsequent vacuum impregnation of EP. The obtained epoxy composite possesses a high TC of 10.27 W m^−1^K^−1^ at a relatively low bioSiC loading of 21 vol%, corresponding to a significant enhancement efficiency of 259 per 1 vol% loading when compared with the pure EP, indicating the highest value of efficiency among other reported epoxy composites. Meanwhile, a high anisotropic TC ratio of 5.77 is also achieved. These are because the wood tissue anisotropic microstructure is completely replicated in the bioSiC framework, and the vertically aligned dense SiC microchannels provide an efficient pathway for the heat transfer. Furthermore, the bioSiC/EP composite also presents an extremely low CLTE of 12.23 ppm K^−1^_,_ a high thermal stability, and an excellent flame resistance. These are because the 3D bioSiC framework possesses a barrier effect, which can restrict the EP thermal expansion and retard the EP degradation. Furthermore, high flexural strength of 222 MPa could be achieved for the 19‐bioSiC/EP composite, due to the strong 3D interconnected bioSiC framework. Our work provides a new insight into the design and fabrication of thermal management materials with high performance for versatile applications in advanced electronic packaging fields to address the heat dissipation.

## 4 Experimental Section

### Materials

Natural poplar wood was purchased from a furniture factory in Guiyang, China. SiO powders with a purity of 99% and *β*‐SiC powders were purchased from Shanghai Chaowei Nano Technology Co., Ltd, China. 3‐glycidoxypropyltrimethoxy silane (KH‐560) as a silane coupling agent was obtained from Shanghai Aladdin Biochemical Technology Co., Ltd, China. Bisphenol‐A EP was offered by Shanghai Resin Factory Co., Ltd, China. Methyltetrahydrophthalic anhydride (MeTHPA) and 2,4,6‐tris(dimethylaminomethyl)phenol (DMP‐30) were purchased from Sinopharm Chemical Reagent Co., Ltd, China, which were used as curing agent and curing accelerator, respectively.

### Preparation of Pyrolytic Carbon Template

Rectangular specimens in both the axial (||) and radial (⊥‐1, ⊥‐2) orientations with dimensions of 100 mm × 40 mm × 40 mm were cut from natural poplar wood. The specimens were dried in an oven at 110 ℃ for 1 day to remove water before pyrolysis. Subsequently, the dried samples were pyrolyzed at 1000 ℃ for 4 h under nitrogen atmosphere with a low heating rate of 1 ℃ min^−1^, resulting in the intact pyrolytic carbon template.

### Preparation of 3D BioSiC Skeleton

First, the carbon template was machined into various dimensions and shapes in both the axial (||) and radial (⊥‐1, ⊥‐2) orientations. Then the samples and SiO powders were put into a closed graphite crucible, which was put into a sintering furnace (High Multi‐5000, Fujidempa Co., Ltd, Japan) and sintered at 1800 ℃ for 4 h under argon pressure of 0.225 MPa. SiO vapor was generated via sublimation of SiO powders at high temperature, which reacted with the carbon template to form 3D bioSiC skeleton. The heating rate from room temperature to 1200 ℃ was 20 and 5 ℃ min^−1^ for 1200 to 1800 ℃.

### Preparation of BioSiC/EP Composite

The 3D bioSiC scaffold was further infiltrated with EP under vacuum to prepare the bioSiC/EP composite. Before the impregnation, the samples were soaked in a hydrolysis solution (mass ratio of ethanol:water:KH‐560 = 95:5:0.57) of silane coupling agent at 80 ℃ for 3 h for surface modification. After that, the EP along with the curing agent and the accelerator were uniformly mixed at 55 ℃ for 40 min (EP:MeTHPA:DMP‐30 = 100:86:1). Subsequently, the functionalized 3D bioSiC framework was immersed into the resulted mixture under the assistance of vacuum for 4 h to ensure the complete filling. Finally, the composites were cured in an oven at 70 ℃ for 10 min, 135 ℃ for 3 h, and 180 ℃ for 4 h.

### Preparation of Random SiC/EP Composite

In order to be consistent with the bioSiC nanocrystals size, *β*‐SiC powders (average size: 500 nm) were used for the preparation of random SiC/EP composite. First, the dried SiC powders were added to a hydrolysis solution (mass ratio of ethanol:water:KH‐560 = 95:5:0.57) of silane coupling agent and stirred at 80 ℃ for 3 h for surface modification. Subsequently, the modified SiC powders were obtained by vacuum filtration and drying at 80 ℃ for 1 day. Second, the modified SiC powders along with the liquid EP, the curing agent and the accelerator were uniformly mixed at 55 ℃ for 4 h under the assistance of vacuum to ensure no bubbles (SiC:EP:MeTHPA:DMP‐30 = 119:100:86:1). Finally, the random SiC/EP composites with 19 vol% SiC were obtained by curing in an oven at 70 ℃ for 10 min, 135 ℃ for 3 h, and 180 ℃ for 4 h.

### Measurements and Characterizations

The porosity and density of the samples were measured by the drainage method according to the Archimedes principle. The morphology and quantitative elemental analyses of the carbon template, the 3D bioSiC scaffold, and the corresponding epoxy composite were observed by a field emission scanning electron microscopy (FESEM, Zeiss GeminiSEM 500, Germany). FTIR (Bruker VERTEX70, American) was used to characterize the functional groups on the surface of the 3D bioSiC network before and after surface modification. XPS were detected by a Thermo Scientific X‐ray photoelectron spectrometer (Thermo Fisher ESCALAB Xi+, American). XRD analysis of the specimens was performed by an X‐ray diffractometer (PANalytical X'Pert PRO, Netherlands) with Cu K*α* radiation. The thermal diffusivity (*α*) and specific heat capacity (*C*_p_) of the samples were measured by a laser‐flash diffusivity instrument (Netzsch LFA467, Germany) at room temperature, and the eventual TC (*λ*) was calculated by the equation: *λ* = *ρ* × *α* × *C*_p_, *ρ* was the density of the specimens, each data was measured by a specimen, but three measurement points were selected for each specimen, each final value was averaged with three measurements. The temperature distribution image of the samples was recorded by an infrared thermograph (Fotric 285, China). The CLTE of the specimens was tested by a thermomechanical analyzer (Netzsch DIL 402 C, Germany) with a heating rate of 10 ℃ min^−1^ and a preload force of 0.02 N, each data was measured by a specimen. Thermal stability of the bioSiC/EP composite was performed by a TGA (TA SDT Q600, American) at a heating rate of 10 ℃ min^−1^ under an air atmosphere. The flexural strength was measured by a universal testing machine (UTM 2103, China) via three‐point bending method with a 16 mm span at a cross‐head speed of 0.5 mm min^−1^. Each final value was averaged over five measurements.

### Statistical Analysis

In Figure 3e–i, the XPS data were pre‐processed by Avantage 5.52, including charge correction and peak fitting. In Figure 4a–c, each TC data point was obtained from an average of three independent measurements. In Figure 4a,b, except that the data of bioSiC_||_/EP composite was fitted by Foygel model (*K* = *K*0(*V*f − *V*c)^*τ*^ + *C*),^[^ ^56^ ^]^ other data were fitted by linear regression (*K* = *AV*f + *B*), which were analyzed with the goodness of fit (*R* ^2^) and F‐test, statistical significance was defined as probability value (*p*) < 0.05. The CLTE values of pure EP, random SiC/EP, and bioSiC/EP composites in Figure 7a–c were the average CLTE in the temperature range of 30–180 ℃. In Figure 10, each final value was averaged over five measurements. All plotting and analysis were conducted with Origin 2019b. No statistical methods were used to predetermine sample sizes, and the exact values of sample size are provided in the sub‐section entitled “Measurements and Characterizations” at the Experimental Section.

## Conflict of Interest

The authors declare no conflict of interest.

## Supporting information

Supporting Information

## Data Availability

Data available on request from the authors.
