# Supplementary material for: Wood‐Derived, Vertically Aligned, and Densely Interconnected 3D SiC Frameworks for Anisotropically Highly Thermoconductive Polymer Composites
Source: Adv Sci (Weinh). 2022 Jan 13;9(7):2103592. doi: 10.1002/advs.202103592 (PMC8895159; doi:10.1002/advs.202103592)
Supplement: Supplementary file 1 — Supporting Information [file ADVS-9-2103592-s001.pdf]

ADVANCED SCIENCE
Open Access

Supporting Information

for *Adv. Sci.,* DOI: 10.1002/advs.202103592

Wood-Derived, Vertically Aligned and Densely Interconnected 3D SiC Frameworks for Anisotropically Highly Thermoconductive Polymer Composites

Xiaonan Zhou, Songsong Xu, Zhongyu Wang, Liucheng Hao, Zhongqi Shi, Junping Zhao, Qiaogen Zhang, Ishizaki Kozo, Bo Wang, and Jianfeng Yang**

Supporting Information

Wood-Derived, Vertically Aligned and Densely Interconnected 3D SiC Frameworks for Anisotropically Highly Thermoconductive Polymer Composites

Xiaonan Zhou, Songsong Xu, Zhongyu Wang, Liucheng Hao, Zhongqi Shi, Junping Zhao, Qiaogen Zhang, Ishizaki Kozo, Bo Wang, and Jianfeng Yang**

X. Zhou, S. Xu, Z. Wang, L. Hao, Z. Shi, B. Wang, J. Yang
State Key Laboratory for Mechanical Behavior of Materials
Xi'an Jiaotong University
Xi'an 710049, China
E-mail: wangbo_1@xjtu.edu.cn (B. Wang)
E-mail: yang155@.xjtu.edu.cn (J. Yang)

L. Hao, B. Wang
High Voltage Switchgear Insulation Materials Laboratory of State Grid
Pinggao Group Co., Ltd
Pingdingshan 467001, China
E-mail: wangbo_1@xjtu.edu.cn (B. Wang)

J. Zhao, Q. Zhang
State Key Laboratory of Electrical Insulation and Power Equipment
Xi'an Jiaotong University
Xi'an 710049, China

I. Kozo
Department of Mechanical Engineering
Nagaoka University of Technology
Nagaoka 940−2188, Japan

The supporting information file includes:

Figure S1. Microstructures of anisotropic carbon template and bioSiC ceramic. Top-view SEM images of a) carbon template and d) bioSiC ceramic. Left-view SEM images of b) carbon template and e) bioSiC ceramic. Main-view SEM images of c) carbon template and f) bioSiC ceramic.

Equation S1. Foygel model.

Equation S2. Contact resistance (R).

Table S1. Comparison of R of our work with other epoxy composites reported in previous work.

Table S2. Comparison of anisotropic TC ratio of our work with other epoxy composites reported in previous work.

Table S3. Comparison of TC and η of our work with other epoxy composites reported in previous work.

Figure S2. The dynamic variations of the TC models. a) pure EP, b) bioSiC$_{||}$/EP composite, c) bioSiC$_{\perp}$/EP-1 composite, d) bioSiC$_{\perp}$/EP-2 composite.

Equation S3. ROM model.

Equation S4. Turner model.

Equation S5. Kerner model.

Table S4. The average displacement of each surface of the models.

Table S5. The thermal stability information of pure EP and bioSiC/EP composites.

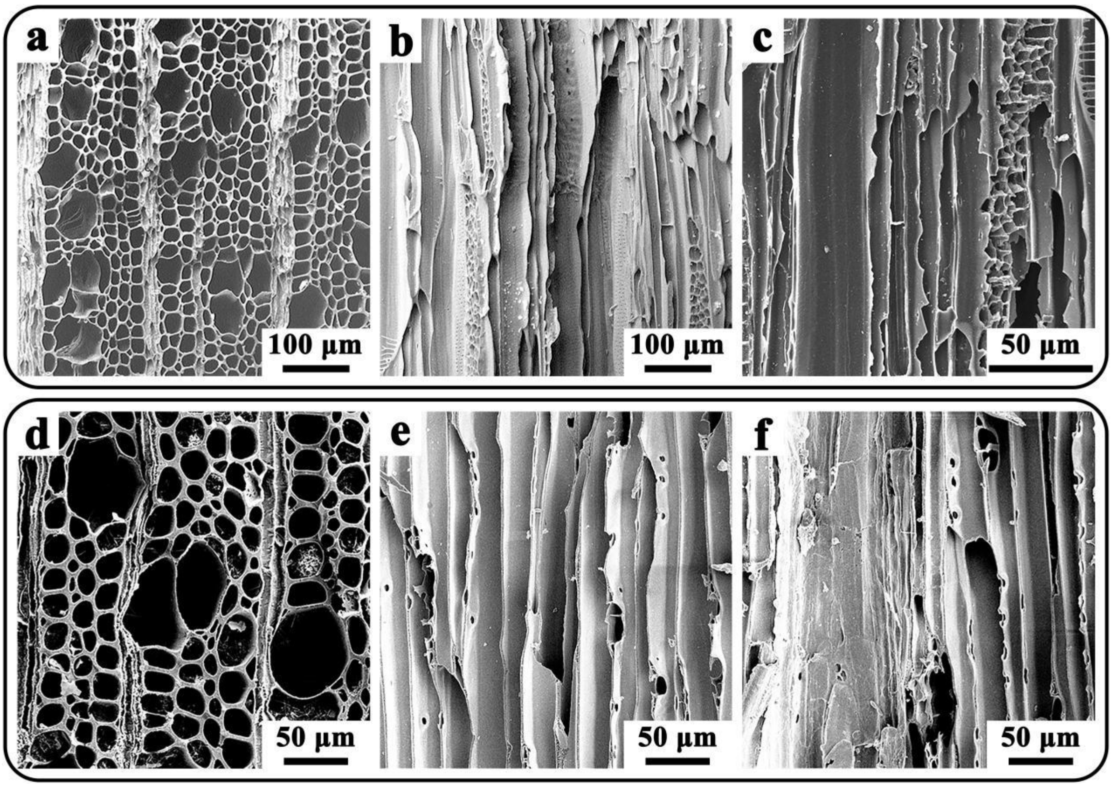

Figure S1. Microstructures of anisotropic carbon template and bioSiC ceramic. Top-view SEM images of a) carbon template and d) bioSiC ceramic. Left-view SEM images of b) carbon template and e) bioSiC ceramic. Main-view SEM images of c) carbon template and f) bioSiC ceramic.

Equation S1. Foygel model.[1]

$$K = K_0(V_f - V_c)^{\tau} + C \qquad \text{(S1)}$$

Equation S2. Contact resistance (R).

$$R = (K_0 V_c^{\tau} L)^{-1} \qquad \text{(S2)}$$

where K_0 is a preexponential factor ratio that is related to the expected contribution of the filler network alone. V_c is the critical volume fraction at the thermal percolation threshold. τ is a conductivity exponent that is dependent on the aspect ratio of the filler. C is an adjustment constant and L is the average size of the SiC nanocrystals.

Table S1. Comparison of R of our work with other epoxy composites reported in previous work.

Sample	K_0 (W m^{-1} K^{-1})	V_c (%)	τ	L (nm)	R (K W^{-1})	Year	References
3D	72	5.00	1.65	300	6.49E+06	2015	[2]
BNNS/EP	55	5.00	1.65	300	8.50E+06	2015	[2]
BNNSs/Ag NPs/EP	38	1.00	–	200	4.00E+08	2016	[3]
BNNSs/EP	15	1.00	–	200	8.00E+08	2016	[3]
bioSi$C_{\parallel}$/EP	370	9.70	1.93	700	3.49E+05	Our work	

Table S2. Comparison of anisotropic TC ratio of our work with other epoxy composites reported in previous work.

Sample	Filler loading (vol%)	$TC_{\parallel}$ (W m^{-1} K^{-1})	$TC_{\perp}$ (W m^{-1} K^{-1})	anisotropic TC ratio	Year	References
GHF/EP	19	8.43	3.36	2.51	2018	[4]
MLG/EP	12 wt%	33.54	7.55	4.44	2014	[5]
GNP/EP	11 wt%	4.50	0.90	5.00	2013	[6]
MWCNT/EP	17	4.87	1.00	4.87	2011	[7]
Natural Wood	–	0.15	0.10	1.50	2018	[8]
Wood Aerogel	–	0.12	0.03	4.30	2018	[8]
3D Si_3N_4NW/EP	60	9.20	5.70	1.61	2013	[9]
AlN-H/EP	47	9.48	4.45	2.13	2020	[10]
3D BNNS/EP	9	2.85	2.40	1.19	2015	[2]
BN/EP	15	5.19	3.48	1.49	2018	[11]
h-BN/EP	20 wt%	–	–	2.04	2013	[12]
MA BNNS/EP	10	–	–	2.53	2013	[13]
Vertical BN-SiC/EP	40	5.77	2.25	2.56	2016	[14]
SiCF Network	6	0.65	0.54	1.20	2016	[15]
3D SiCNW/EP	2	1.67	1.45	1.15	2018	[16]
bioSiC/EP	21	10.27	1.78	5.77	Our work	

Table S3. Comparison of TC and η of our work with other epoxy composites reported in previous work.

Sample	Filler loading (vol%)	TC ($W\ m^{-1}\ K^{-1}$)	η	Year	References
Metallic wood	31	5.23	-	2017	[17]
AgNW/EP	4	0.90	78	2018	[18]
AgNW/GP-DOPO/EP	4	1.41	136	2018	[18]
Ni powder/EP	30	2.14	20	1998	[19]
Al particle/EP	48	1.47	12	2010	[20]
GHF/EP	19	8.43	209	2018	[4]
GNP/EP	25	6.44	124	2007	[21]
G/MLG/EP	10	5.10	244	2012	[22]
MWCNT/EP	17	4.87	106	2011	[7]
RGO/(f)SWCNT/EP	4	0.63	80	2020	[23]
3D-BNNS/EP	4	1.56	167	2019	[24]
3D-C-BNNS/EP	10	3.13	161	2017	[25]
3D BN-rGO/EP	13	5.05	206	2018	[26]
3D-BNNS/EP	9	2.85	181	2015	[2]
BN/EP	15	5.19	166	2018	[11]
Si_3N_4/EP	22	3.89	75	2016	[27]
β-Si_3N_4/EP	63	7.08	30	2013	[28]
Si_3N_4NW/EP	60	9.20	75	2013	[9]
AlN-H/EP	47	9.48	72	2020	[10]

Magnetic aligned AlN-Fe_3O_4/EP	40	3.14	37	2016	[29]
Al_2O_3/EP	25	3.01	36	2017	[30]
Al_2O_3/AgNPs/EP	25	6.71	85	2017	[30]
3D-Al_2O_3/EP	43	4.36	34	2019	[31]
Al_2O_3/EP	70	13.46	105	2016	[32]
Al_2O_3/EP	36	3.17	39	2019	[33]
SiC/CF/EP	28	1.23	18	2013	[34]
Magnetic aligned SiC/EP	40	1.87	21	2015	[35]
Magnetic aligned SiC-Fe_3O_4/EP	40	3.15	37	2015	[35]
Vertical BN-SiC/EP	40	5.77	78	2016	[14]
bioSi$C_{\perp}$/EP-2	21	1.78	40		
bioSi$C_{\perp}$/EP-1	21	6.16	152	Our work	
bioSi$C_{\parallel}$/EP	21	10.27	259		

Figure S2. The dynamic variations of the TC models. a) pure EP, b) bioSiC$_{\parallel}$/EP composite, c) bioSiC$_{\perp}$/EP-1 composite, d) bioSiC$_{\perp}$/EP-2 composite.

See the gif files.

Equation S3. ROM model.

$$\alpha_c = V_r \alpha_r + V_m \alpha_m \tag{S3}$$

Equation S4. Turner model.[36]

$$\alpha_c = \frac{\alpha_r K_r V_r + \alpha_m K_m V_m}{K_r V_r + K_m V_m} \tag{S4}$$

Equation S5. Kerner model.[37]

$$\alpha_c = V_r \alpha_r + V_m \alpha_m + V_r V_m \frac{(\alpha_r - \alpha_m)(K_r - K_m)}{K_r V_r + K_m V_m + \frac{3 K_r K_m}{4 G_m}} \tag{S5}$$

where α, V, K and G represent the CLTE, volume fraction, bulk modulus and shear modulus, and the subscripts c, r, m refer to the composite, reinforcement and matrix, respectively.

Table S4. The average displacement of each surface of the models.

Model	Direction	Surface	Displacement (μm)	Size (μm)	Linear expansivity (%)
A single unit	x	left	-0.000461	22	0.004129
		right	0.000447		
	y	front	-0.000470	22	0.004129
		back	0.000438		
	z	bottom	-0.000523	22	0.004877
		top	0.000550		
Multiple units	x	left	-0.003388	384	0.001770
		right	0.003410		
	y	front	-0.003733	384	0.001941
		back	0.003718		
	z	bottom	-0.004363	384	0.002316
		top	0.004530		

Table S5. The thermal stability information of pure EP and bioSiC/EP composites.

Sample	$T_{10\%}$ (℃)	$T_{50\%}$ (℃)	Residual weight (wt%)	Maximum decomposition rate of first stage (wt% ℃$^{-1}$)	Temperature at the maximum decomposition rate of first stage (℃)	Maximum decomposition rate of second stage (wt% ℃$^{-1}$)	Temperature at the maximum decomposition rate of second stage (℃)
Pure EP	360	406	0.00	1.86	395	0.32	559
17-bioSiC/EP	376	547	40.07	0.72	402	0.25	561
18-bioSiC/EP	382	543	42.08	0.96	405	0.21	551
19-bioSiC/EP	386	548	43.80	0.74	414	0.18	551

References

[1] M. Foygel, R. D. Morris, D. Anez, S. French, V. L. Sobolev, Phys. Rev. B. **2005**, 71, 104201.

[2] X. Zeng, Y. Yao, Z. Gong, F. Wang, R. Sun, J. Xu, C. P. Wong, Small. **2015**, 11, 6205.

[3] F. Wang, X. Zeng, Y. Yao, R. Sun, J. Xu, C. P. Wong, Sci. Rep. **2016**, 6,19394.

[4] F. An, X. Li, P. Min, P. Liu, Z. G. Jiang, Z. Z. Yu, ACS Appl. Mater. Interfaces. **2018**, 10, 17383.

[5] Q. Li, Y. Guo, W. Li, S. Qiu, C. Zhu, X. Wei, M. Chen, C. Liu,S. Liao, Y. Gong, A. K. Mishra, L. Liu, Chem. Mater. **2014**, 26, 4459.

[6] X. Tian, M. E. Itkis, E. B. Bekyarova, R. C. Haddon, Sci. Rep. **2013**, 3, 1710.

[7] A. M. Marconnet, N. Yamamoto, M. A. Panzer, B. L. Wardle, K. E. Goodson, ACS Nano. **2011**, 5, 4818.

[8] J. Song, C. Chen, Z. Yang,Y. Kuang,T. Li, Y. Li, H. Huang, I. Kierzewski, B. Liu, S. He, T. Gao, S. U. Yuruker, A. Gong, B. Yang, L. Hu, ACS Nano. **2018**,12, 140.

[9] T. Kusunose, T. Yagi, S. H. Firozc, T. Sekinod, J. Mater. Chem. A. **2013**, 1, 3440.

[10] Z. Wei, W. Xie, B. Ge, Z. Zhang, W. Yang, H. Xia, B. Wang, H. Jin, N. Gao, Z. Shi, Compos Sci Technol. **2020**, 199, 108304.

[11] Z. Tian, J. Sun, S. Wang, X. Zeng, S. Zhou, S. Bai, N. Zhao, C. P. Wong, J. Mater. Chem. A. **2018**, 6, 17540.

[12] Z. Lin, Y. Liu, S. Raghavan, K. Moon, S. K. Sitaraman, C.P. Wong, ACS Appl. Mater. Interfaces. **2013**, 5, 7633.

[13] H. B. Cho, M. Mitsuhashi, T. Nakayama, S. Tanaka, T. Suzuki, H. Suematsu, W. Jiang, Y. Tokoi, S. W. Lee, Y. H. Park, K. Niihara, Mater Chem Phys. **2013**, 139, 355.

[14] K. Kim, H. Ju, J. Kim, Compos Sci Technol. **2016**, 123, 99.

[15] C. Ferraro, E. Garcia-Tuñon, V. G. Rocha, S. Barg, M. D. Fariñas, T. E. Gomez Alvarez-Arenas, G. Sernicola, F. Giuliani, E. Saiz, Adv. Funct. Mater. **2016**, 26, 1636.

[16] Y. Yao, X. Zhu, X. Zeng, R. Sun, J. B. Xu, C. P. Wong, ACS Appl. Mater. Interfaces. **2018**, 10, 9669.

[17] J. Wan, J. Song, Z. Yang, D. Kirsch, C. Jia, R. Xu, J. Dai, M. Zhu, L. Xu, C. Chen, Y. Wang, Y. Wang, E. Hitz, S. D. Lacey, Y. Li, B. Yang, L. Hu, Adv. Mater. **2017**, 29, 1703331.

[18] Y. Feng, X. Li, X. Zhao, Y. Ye, X. Zhou, H. Liu, C. Liu, X. Xie, ACS Appl. Mater. Interfaces. **2018**, 10, 21628.

[19] S. Nikkeshi, M. Kudo, T. Masuko, J. Appl. Polym. Sci. **1998**, 69, 2593.

[20] W. Zhou, D. Yu, J. Appl. Polym. Sci. **2010**, 118, 3156.

[21] A. Yu, P. Ramesh, M. E. Itkis, E. Bekyarova, R. C. Haddon, J. Phys. Chem. C. **2007**, 111, 7565.

[22] K. M. F. Shahil, A. A. Balandin, Nano Lett. **2012**, 12, 861.

[23] X. Liang, F. Dai, ACS Appl. Mater. Interfaces. **2020**, 12, 3051.

[24] X. Wang, P. Wu, ACS Appl. Mater. Interfaces. **2019**, 11, 28943.

[25] J. Chen, X. Huang, Y. Zhu, P. Jiang, Adv. Funct. Mater. **2017**, 27, 1604754.

[26] Y. Yao, J. Sun, X. Zeng, R. Sun, J. B. Xu, C. P. Wong, Small. **2018**, 14, 1704044.

[27] L. Yin, X. Zhou, J. Yu, H. Wang, C. Ran, Composites, Part A. **2016**, 90, 626.

[28] Y. Zhu, K. Chen, F. Kang, Solid State Commun. **2013**, 158, 46.
[29] K. Kim, J. Kim, Composites, Part B. **2016**, 93, 67.
[30] G. Pan, Y. Yao, X. Zeng, J. Sun, J. Hu, R. Sun, J. B. Xu, C. P. Wong, ACS Appl. Mater. Interfaces. **2017**, 9, 33001.
[31] L. C. Hao, Z. X. Li, F. Sun, K. Ding, X. N. Zhou, Z. X. Song, Z. Q. Shi, J. F. Yang, B. Wang, Composites, Part A. **2019**, 127, 105648.
[32] Y. Hu, G. Du, N. Chen, Compos Sci Technol. **2016**, 124, 36.
[33] Y. Wu, K. Ye, Z. Liu, B. Wang, C. Yan, Z. Wang, C. T. Lin, N. Jiang, J.Yu, ACS Appl. Mater. Interfaces. **2019**, 11, 44700.
[34] A. Ma, H. Li, W. Chen, Y. Hou, Polym Plast Technol Eng. **2013**, 52, 295.
[35] K. Kim, M. Kim, J. Kim, J. Kim, Ceram. Int. **2015**, 41,12280.
[36] P. S. Turner, J. Res. Natl. Bur. Stand. **1946**, 37, 239.
[37] E. H. Kerner, Proc. Phys. Soc. B. **1956**, 69, 808.
